# Herbal and Dietary Supplements as Adjunctive Treatment for Mild SARS-CoV-2 Infection in Italy

**DOI:** 10.3390/nu17020230

**Published:** 2025-01-09

**Authors:** Anna Licata, Aurelio Seidita, Silvia Como, Gabriele de Carlo, Marcella Cammilleri, Roberta Bonica, Maurizio Soresi, Nicola Veronese, Roberta Chianetta, Roberto Citarrella, Lydia Giannitrapani, Mario Barbagallo

**Affiliations:** 1 Unit of Internal Medicine, AOU Policlinico “P. Giaccone”, Department of Health Promotion Sciences, Maternal and Infant Care, Internal Medicine and Medical Specialties (PROMISE), University of Palermo, 90134 Palermo, Italy; silvia.como29@gmail.com (S.C.); marcella.cammilleri@gmail.com (M.C.); nicola.veronese@unipa.it (N.V.);; 2 Unit of Internal Medicine, “V. Cervello” Hospital, Ospedali Riuniti “Villa Sofia-Cervello”, 90146 Palermo, Italy; aurelio.seidita@unipa.it (A.S.);; 3 Department of Health Promotion Sciences, Maternal and Infant Care, Internal Medicine and Medical Specialties (PROMISE), University of Palermo, 90146 Palermo, Italy; 4 Institute for Biomedical Research and Innovation (IRIB), National Research Council (CNR), 90146 Palermo, Italy

**Keywords:** essential oils, *Echinacea*, ginseng, vitamin C, L-arginine, vitamin D, COVID-19

## Abstract

During the COVID-19 pandemic, several observational studies proved a certain efficacy of nutraceuticals, herbal products, and other dietary supplements as adjuvant therapies used alongside antiviral drugs. Although their use has not been widespread in Italy, according to preliminary evidence, many supplements with demonstrated immunomodulatory effects, such as vitamins C and D, herbal medicines and essential oils, might relieve the respiratory symptoms of COVID-19, since SARS-CoV-2 can activate inflammasome-mediated inflammatory signaling pathways. Other observational studies have shown that herbal treatments, such as *Echinacea purpurea* and ginseng, help alleviate respiratory symptoms and reduce serum levels of inflammatory cytokines, which are typically overexpressed in both adult and pediatric SARS-CoV-2 patients. Further, vitamins C and D can attenuate the immune response thanks to their cytokine suppression ability and to their known antimicrobial activity and potential to modulate T helper cell response. The strong immune response triggered by SARS-CoV-2 infection is responsible for the severity of the disease. Preliminary data have also shown that L-arginine, an endothelial-derived relaxing factor, is able to modulate endothelial damage, which appears to be one of the main targets of this systemic disease. Finally, some essential oils and their isolated compounds, such as eucalyptol, may be helpful in reducing many of the respiratory symptoms of COVID-19, although others, such as menthol, are not recommended, since it can lead to an undervaluation of the clinical status of a patient. In this narrative review, despite the lack of strong evidence in this field, we aimed to give an overview of the current available literature (mainly observational and cross-sectional studies) regarding herbal products and dietary supplements and their use in the treatment of mild disease from SARS-CoV-2 infection. Obviously, dietary supplements and herbal products do not constitute a standardized treatment for COVID-19 disease, but they could represent an adjunctive and useful treatment when used together with antivirals.

## 1. Introduction

Since the beginning of the COVID-19 pandemic, many non-pharmacological therapeutic approaches, such as herbal supplements and dietary therapies, in addition to the standardized treatments, have been analyzed. Most patients with SARS-CoV-2 infection have mild-to-moderate upper respiratory tract symptoms and receive outpatient setting care. In this scenario, preliminary studies with limited clinical data, have tested “natural” approaches, hypothesizing that the early use of many substances with a proven immune-stimulating role, such as essential oils, ginseng and *Echinacea*, as well as vitamins C and D, may improve prognosis and disease course [1].

The increasing attention to such immunomodulatory and anti-inflammatory products is primarily related to their wide availability in the form of dietary supplements. Food consumption habits appear to have changed during the COVID-19 pandemic, mainly based on suggestions by health professionals, including pharmacists and herbalists, and also on information available online [1]. The latter, due to their ease of availability and not always traceable origin, should always be carefully checked, since, even with the best purposes, they could mislead the patient as well as non-specialized personnel. In this scenario, many herbs have been reported to relieve upper respiratory tract infection symptoms, so that, during the COVID-19 pandemic, their use greatly increased, especially in the management of patients with mild disease who did not require hospitalization, and, sometimes, in addition to standardized treatments.

Despite the absence of high-quality evidence, herbs have been used to alleviate complications associated with COVID-19, as an adjunctive therapy to currently approved drugs. Eucalyptol and other essential oils can have an antimicrobial and anti-inflammatory effect, by downregulating inflammatory mediators (TNF-α, IL-6, and IL-8) through epigenetic modifications within the white blood cells [2]. Many herbs such as ginseng have shown an anti-atherosclerotic and antithrombotic function, as well as an antiviral and immune-stimulating role, and, consequently, their efficacy has been tested in SARS-CoV-2 infection [3]. *E. purpurea* extracts from roots and leaves have shown to strongly modulate the non-specific immune response, by improving the oxidative capacity of cells and activating natural-killer cells and T cells. *E. purpurea* extracts have been found to inhibit, in in vitro studies, both coronavirus replication, including SARS-CoV-2, and inflammatory cytokine production [4,5].

Many studies proved a significant vitamin D deficiency in COVID-19 patients [6,7,8]. The known immunomodulatory activities of this vitamin [9,10,11,12,13,14] seem to be able to decrease the production of pro-inflammatory cytokines, which are typically overexpressed in SARS-CoV-2-infected patients.

In the case of vitamin C, its antiviral [15,16] and antioxidant effects via the attenuation of immune response activation have already been known for some time [17,18], even managing to promote the reduction in hospital mortality in patients with sepsis [19], even if with contrasting results. Therefore, intravenous high-dose vitamin C treatment has been used in COVID-19 patients.

L-arginine has been used to increase vasodilation due to its ability to generate nitric oxide (NO), which has been shown to be the most important endothelial relaxation factor [20,21,22,23]. This function seems to be useful in patients with severe SARS-CoV-2 infection, since endothelium is a primary target of the disease. All these reasons provide strong motivations for these dietary supplements to be considered as feasible non-pharmacological therapeutic choices in the management of mild COVID-19 disease.

Despite reports of a positive effect linked to the intake of natural products in subjects affected by COVID-19, as well as other pathologies, it should not be forgotten that an indiscriminate use of such products can also determine adverse effects. An incautious use often at excessive doses can compromise the health of the patient, both by direct toxicity and by pharmacological interactions, as reported for many natural substances as ginseng [24] and others [25,26].

In this narrative review, we aimed to give an impartial report of the current available literature regarding herbal products and dietary supplements and their use in the treatment of mild disease from SARS-CoV-2 infection. However, the reader should be aware before entering this narrative review that the works we cite often concern only in vitro evidence and that in vivo studies on both animal models and humans often lack suitable standardization criteria to make them high-quality evidence.

## 2. Herbal and Dietary Supplements as Modulators of Inflammatory Response: From Biochemical Actions to Their Role in Respiratory Tract Diseases

Vitamins, herbs, and nutraceutical immunomodulatory and anti-inflammatory activities have been extensively used to enhance validated treatments of infective, metabolic, cardiovascular, and neoplastic diseases [27,28,29]. Given these well-known capabilities and the wide availability on the market of products containing substances such as vitamin C, vitamin D, ginseng and other natural herbs, during the COVID-19 emergency, many researchers tried to analyze whether the use of these products could be effective in managing symptoms related to SARS-CoV-2 infection. In this paragraph, we wanted to summarize some of the main abilities of these substances, focusing attention on the main infectious and non-infectious diseases that can affect the respiratory system, then focusing in the following paragraph on the evidence, which is fragmentary and often based only on observational studies, in the SARS-CoV-2 infection.

### 2.1. Beneficial Effects of Essential Oils

Essential oils are made up of a mixture of organic terpenes, terpenoids, phenylpropanoids and aldehydes, which enter the bloodstream via the respiratory system (i.e., aromatherapy, the practice of inhaling essential oils) and may activate the limbic system, affecting emotions and behavior.

Their positive effects, although with limited evidence and based mainly on in vitro studies or small clinical trials, have been proven in several virus-induced diseases such as human immunodeficiency virus (HIV) [30,31], influenza [32,33,34], human herpes viruses [35,36,37], avian influenza [38,39], and yellow fever virus [40,41], as well as in asthma [42,43,44] and obstructive pulmonary disease [45,46]. Eucalyptus oil has been used over the years to treat many respiratory disorders, such as bronchitis, pharyngitis, and sinusitis, through the action of its active constituent, 1,8-cineole, which has been shown to have muscle relaxant effects by reducing the smooth muscle contractions of the airways [47]. Moreover, the inhalation of cineole has a proven anti-inflammatory and analgesic effect by blocking cytokine release [48].

In addition, many essential oil compounds have shown anti-viral activities by interacting with key protein targets. Eucalyptus and thymus oils suppress the main external proteins of both the influenza virus and herpes simplex virus type 1 (HSV-1), whereas clove and oregano oils could inhibit the poliovirus, coxsackievirus B1, and adenovirus type 3 [49,50]. Eugenol, a monoterpenoid of clove essential oil, also exhibits an HSV virucidal effect [51].

Finally, some studies have also shown that these substances may disrupt the viral envelope [52].

### 2.2. Ginseng’s Medical Properties

Ginseng is a medicinal plant, originating from North America and East Asia, used to improve both physiological conditions and diseases. The three most common species in use are American ginseng, Asian (or Korean) ginseng, (Panax quinquefolius and Panax ginseng, respectively), and *Siberian ginseng*, also called “*eleuthero*” (*Eleutherococcus senticosus*).

Ginseng’s medical properties have been known for centuries, and several of its active compounds (e.g., ginsenosides, polysaccharides, phytosterols, essential oils, glycosides, and saponins) have shown activity against inflammatory [53,54], autoimmune [55,56,57], cardiovascular [58,59,60], metabolic diseases [61,62,63,64], and cancer [65,66].

The most active constituents of ginseng are saponin glycosides, which are glycosylated steroids named ginsenosides. In vitro and in vivo studies [67] have reported their immune-stimulating and anti-tumoral activities: improving cardiovascular function through vasodilation and the reduction in platelet aggregation; antioxidant activity through an increased elimination of oxygen free radicals and decreased lipid peroxidation; and stimulation of the adrenocortical system and hypoglycemic activity. Many ginsenosides protect cell membranes, especially those of the immune and nerve cells. An in vivo study has shown a reduction in influenza virus infection in volunteers treated with 100 mg of a standardized ginseng extract G115, probably via the activation of natural killer and T-cells [68]. Few adverse effects were found; the main one was insomnia. A recent meta-analysis of randomized control trials (RCTs) [67] also showed that ginseng may effectively reduce mental and physical fatigue as it increases cortical levels of noradrenalin, dopamine, cyclic adenosine monophosphate (cAMP), and serotonin, which have been considered responsible for mental fatigue. An in vitro study also reported that ginseng boosts the brain’s aerobic metabolism. Ginsenosides stimulate the hypothalamic–pituitary–adrenocortical axis, increasing hemoglobin levels, enhancing myocardial metabolism, promoting vasodilation and consequently oxygen extraction and mitochondrial metabolism in the muscle, all of which could theoretically enhance physical performance and thus improve resistance to exercise stress [67,69].

### 2.3. Properties of Echinacea Purpurea

Herbal medicines derived from Echinacea species have been used for their known antimicrobial properties for several centuries before the introduction of Western medicine [70]. Indeed, several families of viruses such as rhinoviruses [71], influenza viruses [72,73], coronaviruses [74,75], caliciviruses [76], respiratory syncytial viruses [77], herpes viruses [78,79] and polioviruses [80] have been demonstrated to be sensitive to Echinacea extracts. The main viral targets are probably virions, enzymes involved in viral replication, and structures involved in cell adhesion or the entry and egress of virus progeny from infected cells. It has also been reported that *E. Purpurea* extracts reverse the stimulation of pro-inflammatory cytokines IL-1, IL-6, IL-8, and TNFα induced by HSV-1, rhinoviruses, influenza A virus, adenovirus types 3 and 11, respiratory syncytial virus [81], and coronaviruses, thus reducing viral load and the risk and intensity of a cytokine storm. As for other natural products, most of this evidence has been demonstrated in preclinical studies, while the evidence in the clinical field is limited to preliminary studies, mainly observational and in the absence of well-structured clinical trials, consequently limiting a clear clinical recommendation.

### 2.4. L-Arginine and Its Pathophysiological Role

L-arginine is a semi-essential cationic amino acid with a key role in regulating the function of immune and vascular cells.

In addition to being involved in protein synthesis and the removal of ammonia from the urea cycle in the liver, it is the precursor of several molecules, including citrulline, proline, glutamate, homoarginine, polyamines, creatinine, agmatine, and nitric oxide (NO) [20,21,22,23,82]. Since L-arginine is the substrate used to produce NO from nitric oxide synthase (NOS), it seems to have a role in endothelial regulation, acting as an endothelial relaxation factor. Three distinct isoforms of NOS have been identified: neuronal NOS (nNOS or NOS1), inducible NOS (iNOS or NOS2), and endothelial NOS (eNOS or NOS3). iNOS is a calcium-insensitive protein induced by pro-inflammatory cytokines and associated microbial products. When iNOS is stimulated, it generates large amounts of NO with a lifetime. The NO generated by iNOS plays a key role in host defense, mediating a cytotoxic effect on bacteria, parasites, viruses, and tumor cells [83]. NO also seems to be responsible for hypotension in septic shock, since it inhibits arterial tone. Furthermore, the luminal release of NO is known to inhibit coagulation by inducing a powerful anti-thrombotic effect [84], thanks to its action on platelet aggregation, and limiting the intimal thickening of blood vessels by inhibiting the proliferation, migration, and extracellular growth of vascular smooth muscle cells [83,85]. In addition, endothelial cell-derived NO inhibits inflammation by interfering with inflammatory cytokine and chemokine synthesis and recruitment and with the activation of leukocytes in blood vessels. Moreover, it seems that arginine could be used as a substrate from macrophages for the activation of the inflammatory cascade, exerting an antiviral effect [86], and that T-cell function is dependent on its level [87].

From all these premises, it can be deduced that the NO produced by iNOS has a role in the pathophysiology of inflammatory disease and in the severity of clinical presentation during infectious processes, and that arginine, as its substrate, plays an essential role in turn [88].

### 2.5. Actions of Vitamin C

Vitamin C is a water-soluble vitamin and an essential nutrient with an antioxidant action [89]. It has a well-known antioxidant action and works as a cofactor of various biosynthetic pathways in the immune system, also maintaining the skin’s epithelial barrier. Consequently, it has been indicated as a substance capable of reducing mortality in patients in septic shock [15,16,17,18,19], even with contrasting evidence, as recently proven in a systematic review with meta-analysis [90]. Although its role in respiratory tract infections is still controversial and under investigation, it has been widely used in patients with viral respiratory tract infections, including COVID-19 [91].

Vitamin C has been proven to influence the chemotaxis process of neutrophils and lymphocytes as well as the phagocytosis of microbes, protecting the immune cells from the damage occurring after their oxidative burst and activating a caspase-dependent cascade that promotes apoptosis, simultaneously inhibiting necrosis [92,93]. Another feature of vitamin C is its ability to act as an immunomodulatory substance [94], reducing pro-inflammatory cytokine production through the modulation of the nuclear transcription factor kappa B (NF-κB) [95]. In addition, Vitamin C is also able to increase epithelial barrier function, enhance the growth and function of both innate and acquired immune cells, and modulate antibody production [96].

Finally, some direct antiviral mechanisms have been proven, mainly in in vitro studies: the damage of viral capsid due to the redox capacity, and the inhibition of viral replication by degradation of the viral RNA and DNA genomes [97].

### 2.6. The Immune Regulatory Role of Vitamin D

In humans, vitamin D, a steroid hormone, is synthesized via the transformation of 7-dehydrocholesterol after skin exposure to ultraviolet B rays from sunlight; a minor fraction comes from the dietary intake of dairy and fish oils. Beyond its well-known role in bone metabolism, vitamin D receptor (VDR) activation, involving the vitamin D receptor elements (VDREs), regulates the expression of various immune-modulatory genes, like defensins and cathelicidins, and is linked to dendritic cell, macrophage, and neutrophil activation, thus improving mucosal defense [98]. Vitamin D is also known to influence toll-like receptor expression and suppress the adaptive immune response during viral respiratory infections by stimulating a shift in differentiation from Th1 and Th17 towards Th2 and anti-inflammatory regulatory T-cell (Treg) populations. In this scenario, vitamin D may reduce levels of pro-inflammatory cytokines, such as IL-1, IL-6, IL-12, IL-17, and TNF-α, leading both to a lower acquired immune response to viruses and to a lower risk of immune-mediated organ damage [99,100]. Moreover, this vitamin can produce antimicrobial peptides [9] and enhance the production of proteins for tight [10], adherens [11], and gap junctions [12].

Thus, vitamin D has an established immune regulatory role modulating pro-inflammatory cytokine production, T-cell shift differentiation, and Th1 cell pathway suppression; it increases cell immunity by decreasing the cytokine storm [101] and regulating adaptive immunity through the inhibition of Th1 response and the stimulation of CD4+ cell proliferation [102]. Vitamin D has demonstrated activity on the lung parenchyma, with protective effects in interstitial pneumonia in experimental mouse models [103]. Several in vitro studies have shown that vitamin D is able to influence local “respiratory homeostasis”, playing a role in the expression of antimicrobial peptides and downregulating the replication of respiratory viruses [104]. In addition, vitamin D also induces the production of the vascular endothelial growth factor (VEGF) in vascular smooth muscle cells, thus promoting endothelial repair. In endothelial cells, in addition to inducing a reduced expression of inflammatory cytokines, it reduces thrombogenicity and increases endothelial NO production [105]. Thus, vitamin D insufficiency may be involved in acute respiratory distress syndrome (ARDS) and heart failure [101].

## 3. Focus on Natural Product Effects and Possible Activity Pathways in Virus Infection: Is There Any Evidence for SARS-CoV-2?

COVID-19 is primarily an acute respiratory infection, which is often associated with many chronic diseases such as diabetes, kidney impairment, or pulmonary fibrosis, causing a generalized systemic inflammation that may need invasive treatment or long-term medication [106,107,108,109]: therefore, non-invasive interventions should be considered in mild cases to reduce the potential need for advanced treatments or to relieve post-COVID-19 symptoms.

In this scenario, the already reported immunomodulatory and anti-inflammatory features, as well as the efficacy on various types of respiratory viruses, although mostly demonstrated in preclinical studies, of many natural products, could represent an effective adjuvant to the approved management of SARS-CoV-2 infection.

**Figure 1:**
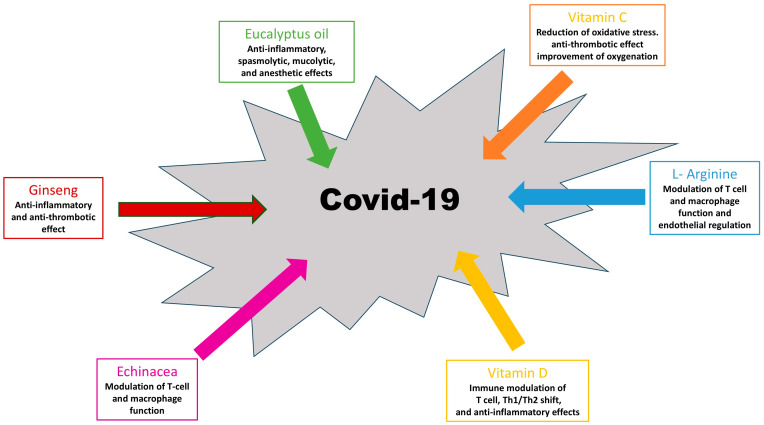
Potential role of herbal and dietary supplements in the management of mild COVID-19.

The action and potential role of herbal and dietary supplements in the management of mild COVID-19 are presented in both Table 1 and Figure 1.

### 3.1. Essential Oils

Essential oil aromatherapy has been little evaluated in patients who recovered from acute COVID-19, since their use in the treatment of acute respiratory symptoms is essentially due to their spasmolytic, anti-inflammatory, anesthetic, and mucolytic effects on both the upper and lower airways. One randomized double blind, placebo-controlled trial evaluated the efficacy of aromatherapy (inhalation of thyme, orange, clove bud, and frankincense) in increasing energy levels in post-COVID-19 syndrome women, proving that the administration of a mixture of essential oils was associated with a reduction in physical and mental fatigue [110]. However, even if essential oils rich in eucalyptol or menthol can be used with due precaution in mild coronavirus infections, including SARS-CoV-2, a recent narrative review showed that high concentrations of menthol are not beneficial in severe COVID-19 patients, as they could reduce dyspnea perception, leading to an underestimation of disease severity [2]. Finally, a recent randomized trial showed how aromatherapy with thyme oil helped in reducing COVID-19 symptoms, such as dizziness, fatigue, headache, loss of appetite, and weakness in hospitalized patients; an effect was also found in hemodynamic parameter regulation, underlining how beneficial the use of this aromatherapy could be when added to pharmacological therapy in these patients [111].

### 3.2. Ginseng

Used as nutraceuticals, ginseng and its constituents (e.g., ginsenosides and saponins) might ameliorate multiple inflammatory diseases by attenuating the priming steps of inflammatory responses, thereby reducing inflammasome activation. SARS-CoV-2 is a condition known to activate inflammasomes, especially in patients with multiple organ involvement due to pyroptotic cell death and cytokine storms, secondary to the increasing production of pro-inflammatory cytokines [112]. Among the inflammasomes, the NLRP3 (nucleotide-binding oligomerization domain, leucine-rich repeat-(NLR) and pyrin domain-containing-3) inflammasome, mainly expressed in the central nervous system, plays a key role in the host immune defense mechanisms against various infectious agents. When activated abnormally in COVID-19 patients, it seems to be linked to some neurological disorders [113], whereas the restriction of inflammasome initiation as well as the improvement of host immunity might have therapeutic benefits. Thus, ginseng and its main active components may provide both preventive and therapeutic effects in COVID-19 patients, both by inhibiting inflammasome activation, and, consequently, hyper-inflammation, and by promoting the host’s antiviral immunity [113].

COVID-19-mediated NLRP3 inflammasome activation in the nervous system is closely related to the severity of several neurological disorders such as stroke and depression. Reducing the level of inflammatory response, including NLRP3 inflammasome activation, may substantially inhibit the proliferation, differentiation, recruitment, and infiltration of resident microglia and peripheral immune cells that induce multi-organ dysfunction, including neurological symptoms. Therefore, the administration of ginseng and its derivates may be considered as an adjuvant therapy in order to counteract the clinical worsening of patients [112,113].

Organ damage caused by COVID-19 frequently includes microvascular and macrovascular thromboembolisms in the spleen, gut, brain, and lungs. Many studies have analyzed the thrombogenic mechanisms in COVID-19, including endothelium inflammation, cytokine storm, disseminated intravascular coagulation (DIC), macrophage activation syndrome (MAS) and overactivation of the renin-angiotensin system (RAS). Many reports have highlighted the action of natural products against thrombosis owing to their anti-platelet activity, suggesting their beneficial use in COVID-19. In this respect, *Panax ginseng’s* potential gene targets have recently been shown to be correlated to the coagulation cascade and to platelet aggregation, and, thus, ginseng might represent a useful supplement for COVID-19 patients, although further studies are needed to validate this [3].

### 3.3. Echinacea Purpurea

*Echinacea purpurea* has a proven activity against enveloped viruses, such as SARS-CoV-2. Preliminary in vitro studies have suggested that a hydroalcoholic extract containing root parts of *E. Purpurea*, named Echinaforce^®^, inhibits MERS- and SARS-CoV-2 infectivity and, in vivo, reduces the concentration of inflammatory cytokines such as TNF-α and IL-1β, increasing at the same time, the cytokine IL-10 [114,115]. This evidence, however, is of limited scale and lacks strong correlation evidence.

A single-center randomized, parallel, open study with a no-treatment control [4] examining the potential of *Echinacea* in preventing and treating respiratory tract infections was carried out in Eastern Europe from November 2020 to May 2021 on 120 healthy volunteers; it was found that, overall, Echinaforce^®^ reduced the viral load and the risk of respiratory tract infections, including SARS-CoV-2, thus representing a possible addition to the existing treatments. Further immunomodulatory mechanisms of *Echinacea* seem to involve the activation of the endocannabinoid system via cannabinoid receptor type 2 (CB2) representing a further approach against cytokine storm. Two recent reviews [116,117] have shown the antiviral and preventive benefits of *Echinacea* in coronavirus infections, which may confirm the possible use of *Echinacea* during COVID-19 infection. In this scenario, a number of studies assessed the impact of *Echinacea* administration 7 days before and 5–7 days after a viral challenge or evaluated its use for 5 to 14 days after a new onset respiratory infection, finding a reduction in symptom severity compared to placebo. On the contrary, other studies reported no differences in disease duration or severity [4,114,116,117].

**Table 1:** Action of natural products and their potential role in COVID-19 therapy.

	Physiological Role	Potential Role in COVID-19 Therapy
Essential oils	Eucalyptus and thyme essential oils suppress external proteins of influenza virus and HSV-1; clove and oregano oils inhibit poliovirus, coxsackie virus B1, and adenovirus type 3 [42,43]. Eugenol has a virucidal effect against human herpes virus [44].	Anti-inflammatory, spasmolytic, mucolytic, and anesthetic effects on the upper and lower airways [43].
	Activation of limbic system pathways [45]	Aromatherapy in long-COVID syndrome [45].
Ginseng	Attenuation of inflammasome activation (including NLRP3) [47].	Inhibition of proliferation, differentiation, recruitment, and infiltration of resident microglia and peripheral immune cells, which induce multi-organ dysfunction, including neurological symptoms, such as stroke and depression [48].
	*Panax ginseng* inhibits endothelium activation and coagulation cascade [3].	Decrease in thrombotic events [3].
*Echinacea purpurea*	Reduction in TNF-α and IL-1β and increase in IL-10 [4].	Reduction in overall viral infections and reduced risk of viral respiratory tract infections [51].
	Activation of the endocannabinoid system via the cannabinoid receptor type 2 [49].	Reduction in cytokine storm [52].
L-Arginine	Protein synthesis; urea cycle; endothelial regulation through NO production [53,54,55,56].	Maintaining immune homeostasis by modulating T-cell and macrophage function [64].
Vitamin C	Increases functionality of mitochondria; influences neutrophil and lymphocyte chemotaxis and phagocytosis of microbes; promotes apoptosis; and modulates NF-κB [75,76,77,78,79].	Reduction in markers of oxidative stress [Carr AC, Galley HF]. Reduction in mortality, thrombosis rate, and improved oxygenation [98,99,100].
Vitamin D	Regulation of expression of various genes like defensins, cathelicidin, linked to dendritic cell, macrophages, and neutrophil activation. Shift from Th1 and Th17 to Th2-type lymphocytes and anti-inflammatory regulatory T-cell differentiation [82,114].	Deficiency is associated with poor prognosis and complications of COVID-19 [115,117,118,119,120,121].

HSV: herpes simplex virus; IL: interleukin; NF-κB: nuclear factor kappa-light-chain-enhancer of activated B-cells; NLRP: nucleotide-binding oligomerization domain, leucine-rich repeat and pyrin domain-containing; NO: nitric oxide; Th: T helper cells; and TNF: tumor necrosis factor.

### 3.4. L-Arginine

Arginine has been widely used against COVID-19, due to its key role in regulating the function of immune and vascular cells [118,119,120].

As previously reported, it represents the precursor of several molecules, including NO. The reduced activity and concentrations of these substances, as occurring during SARS-CoV-2 disease, might cause endothelial dysfunction, the apoptosis of endothelial cells, rupture of the endothelial barrier, increased arterial stiffness, thickening of vessel walls, and an inflammatory and prothrombotic state [121]. When catabolic stress occurs, as happens during infectious processes, endogenous synthesis becomes insufficient to cover metabolic demand [118]. In particular, NO has been shown to interfere with SARS-CoV-2 replication via two distinct mechanisms [122,123]: (1) reducing palmitoylation of the spike protein and, in turn, disrupting interaction between the spike protein and its receptor ACE2; and (2) limiting RNA production in the initial stages of viral replication [124,125,126,127]. However, during the course of SARS-CoV-2 disease, there clearly seems to be a significant risk of ischemia related to vascular disease [126,127].

Arginine administration appears to be useful for maintaining immune homeostasis by modulating T-cell and macrophage function [128]. Patients with COVID-19 have a high amount of myeloid-derived suppressor cells that suppress the proliferation of T-cells and production of IFN-γ through the production of arginase, which metabolizes arginine. This results in T-cell dysfunction and the generation of reactive oxygen species (ROS), which exacerbate inflammation [129,130,131,132]. A recent study analyzing plasma levels of arginine in SARS-CoV-2 patients showed that these are significantly lower than in controls [133]. These low arginine levels appear to correlate with the severity of COVID-19 [77]. Their known role in the expression of the activated GPIIb/IIIa complex translates into platelet activation and thromboembolic events [134].

In fact, a stronger activated GPIIb/IIIa complex expression was found on platelets in patients with severe COVID-19 infection than in healthy subjects and was inversely related to L-arginine plasma concentration. Moreover, RCT studies have demonstrated that L-arginine given with standard care reduces the need for respiratory support and the length of hospitalization, probably related to an increased NO production in SARS-CoV-2 patients. Many in vitro studies have also shown that arginine inhibits the SARS-CoV-2 main protease (M^pro^), which has an extremely relevant role in the life cycle of the virus. This function is intensified when L-arginine is combined with vitamin C [84].

Fiorentino et al. suggested that arginine supplementation resulted in a shorter length of hospital stay and less reliance on respiratory support when compared to patients treated with placebo [135].

### 3.5. Vitamin C

Attention towards vitamin C has arisen from evidence that COVID-19 patients undergo high oxidative stress [136]. In this context, vitamin C can donate electrons and, therefore, protect molecules from this damage. It seems to interfere with mitochondria, which undergo a high degree of oxidative stress and mitophagy during the degenerative processes of COVID-19 [137], preventing the depolarization of their membranes and damage to mitochondrial DNA, which might result in cellular toxicity [138,139].

In COVID-19 disease, the intensity of the inflammatory cascade is responsible for the severity of clinical presentation. Previous studies have already been carried out on intensive care patients with sepsis, pneumonia, multi-organ failure and ARDS, who benefited from this supplementation [140]; similarly, its properties as an immunomodulatory, anti-viral and anti-inflammatory molecule have been proven in several infectious diseases [93,95,141,142]. For all these reasons, there has been considerable interest in supporting the supplementation of this vitamin at high doses in COVID-19 patients [143,144]. Furthermore, it has also emerged that vitamin C supplementation in critical COVID-19 patients improved oxygenation and IL-6 serum levels, although the impact on the mortality rate was poor [145,146].

Neutrophil-derived oxidative stress is thought to induce tissue damage in COVID-19 [147,148], as suggested by the higher oxidative stress marker levels compared to other critically ill patients [149,150]. In these patients, vitamin C may be able to stabilize oxidative stress markers, thus improving survival [15,151]. However, inconclusive data emerged from a recent Cochrane meta-analysis on the therapeutic and preventive effects of vitamin C in pneumonia [152]. In contrast, a reduction in the duration of mechanical ventilation was found in another study [153]. Nevertheless, it should be considered that most of these studies reported single cases or small case-series; some observational studies are available, but clinical studies are scarce or unconvincing. Recently, a systematic review [154] compared nine studies that analyzed the impact of vitamin C supplementation in patients with COVID-19. In 1488 patients suffering from COVID-19, high-dose vitamin C was found to reduce mortality and the thrombosis rate and improve oxygenation [155,156]. However, conflicting results were recorded in other studies where outcomes of patients treated with vitamin C in hospital and outpatient settings [145,157,158] were analyzed, with no impact on mortality, need for mechanical ventilation [159], vasopressor requirements, or SOFA (Sequential Organ Failure Assessment) scores [160].

Vitamin C immunomodulatory properties have also been studied as enhancers of other natural products in treatments of infection, for example, quercetin, which has been hypothesized to interfere at several points of the vital cycle of SARS-CoV-2 (entry, replication and assembly) [161].

Therefore, there does not seem to be sufficient conclusive data to warrant vitamin C supplementation in COVID-19 patients in terms of attenuating susceptibility, progression, and severity of disease, even though the final outcomes could likely have been biased by variables attributable to inclusion criteria in the studies, such as a pre-existing state of hypovitaminosis or comorbidities. Moreover, although many observational studies have shown that low vitamin C levels in patients with severe COVID-19 are associated with a poor prognosis [162,163], and, thus, an intravenous administration of vitamin C monotherapy should be reasonable [16,164,165,166,167], to date, there is insufficient scientific evidence for supplementation in critically ill COVID-19 patients [93,145,168,169].

### 3.6. Vitamin D

The role played by vitamin D on immune system modulating CD4+ cells is particularly relevant during SARS-CoV-2 infection and more specifically on SARS-CoV-2-induced lymphopenia [170]. Observational studies have shown a likely connection between lower levels of vitamin D and the possibility of developing respiratory tract infections, including SARS-CoV-2. In fact, an inverse correlation between vitamin D concentration and COVID-19 cases was analyzed in European subjects in 20 countries, demonstrating an inverse dose–response association between higher vitamin D levels and decreasing risk of hospitalization for COVID-19. Moreover, vitamin D activates many gene expressions involved in the immune response [166]. It has also been reported that a physiological vitamin D concentration may prevent COVID-19 infection by helping to defend the respiratory epithelium from pathogenic invasion, probably through increased defensin and cathelicidin expression levels [171].

Some retrospective studies have shown a 1.5–1.7 times higher probability of testing positive for COVID-19 in patients with vitamin D deficiency. A significant protective effect against respiratory tract infections, including COVID-19, when daily doses of vitamin D were taken, has been reported by meta-analyses based on randomized clinical trials. However, a stronger effect was described in patients with vitamin D deficiency (i.e., serum 25(OH) D < 20 ng/mL) at baseline; similarly, higher mortality rates have been reported in vitamin D-deficient patients compared to those with sufficient vitamin D levels in intensive care units (ICUs), especially in those patients with pre-existent comorbidities such as diabetes, cardiovascular diseases, and obesity. Furthermore, elderly patients with vitamin D deficiency have been reported to have a more severe lung involvement, longer disease duration, and risk of death. A recent study showed a positive association between 25OHD serum levels and the PaO_2_/FiO_2_ ratio, corroborating the correlation between vitamin D deficiency and a lower immune system efficiency in older patients [100,172,173,174,175]. A retrospective cohort study showed that vitamin D supplementation during ICU hospitalization was not useful, but daily vitamin D supplementation [176] before ICU admission seemed to correlate with more ICU-free and ventilator-free days [177]. There are several mechanisms through which vitamin D may protect against respiratory infections, particularly COVID-19, including its action on both immune and non-immune mechanisms. As mentioned above, vitamin D enhances innate immune system functions, inducing macrophages and respiratory epithelial cells to produce several antimicrobial peptides such as cathelicidin LL-37, which destabilizes the bacteria and fungi membranes and respiratory virus envelopes. This latter mechanism was shown by a pilot clinical trial in which a single enteral dose of 400,000 IU vitamin D3 versus placebo was given to septic patients, demonstrating higher serum levels of cathelicidin LL-37 in the treatment group. Moreover, it has been reported that cathelicidin LL-37 competitively binds to the SARS-CoV-2 spike protein, thus preventing a virus—ACE2 receptor interaction. Furthermore, the already mentioned vitamin D down-regulation of Th1 and Th17 cells and the upregulation of Treg cells might shut down the pro-inflammatory cytokine cascade, preventing multi-organ failure and fibrotic changes by activation of the VDR in the pulmonary stellate cells [135,136,137]. In animal models, vitamin D has been proven to suppress renin and, consequently, angiotensin II expression, which is considered to take part in the onset of ARDS, myocarditis, and other major complications observed in COVID-19 patients [178].

Although no conclusive results have been reported about vitamin D supplementation in COVID-19 patients, a growing amount of data suggests the potential benefits of vitamin D levels > 30 ng/mL and preferably at 40 to 60 ng/mL to minimize the risks of COVID-19 [100,174,175,176,177,178,179,180,181,182,183]. Despite the partially discordant results of the multiple studies conducted, a recent systematic review underlined the beneficial role of vitamin D supplementation in COVID-19 infection and its related complications [184]. Sartini et al. analyzed the effects of vitamin D administration in twenty-nine studies (twenty-one randomized clinical trials and eight analytical studies), reporting a significant reduction in intubation rates in randomized clinical trials (OR: 0.50; 95% CI 0.27–0.92) and mortality in analytical studies (OR: 0.45; 95% CI 0.24–0.86). No effects on mortality were proven in randomized clinical trials (OR: 0.80; 95% CI 0.61–1.04). Authors, however, reported different effects of vitamin D administration according to the subgroup analysis, with more pronounced effects in older subjects and severe COVID-19. As an example, randomized clinical trials proved a significant reduction in mortality (OR: 0.58; 95% CI 0.39–0.86) in older patients (>65 years), which was not proven in younger ones (OR: 1.05; 95% CI 0.73–1.53). Authors conclude their meta-analysis: “Discrepancies between randomized clinical trials and analytical studies highlight the need for further large-scale, well-designed trials accounting for baseline vitamin D status, standardized supplementation protocols, and patient characteristics to inform clinical guidelines for vitamin D use in COVID-19 management”. This conclusion is mainly determined by the randomized clinical trial design, which, based on guidelines for pharmaceutical drugs rather than nutrients, often do not find beneficial effects of vitamin D. These studies, in fact, generally enrolled people with relatively high 25(OH)D concentrations, gave relatively low vitamin D doses, analyzed results according to intention to treat rather than baseline, and achieved 25(OH)D concentrations [185].

The aforementioned mechanisms (the lowering of SARS-CoV-2 concentrations and shifting cytokine toward anti-inflammatory effects) seem to indicate that the beneficial effect of vitamin D in SARS-CoV-2 infection could be very time-dependent, so that those who might benefit most from vitamin D administration are subjects who are treated early, or those with 25(OH)D concentrations below about 12 ng/mL or whom 25(OH)D concentrations are increased rapidly either with calcifediol or high-dose vitamin D. In a recent meta-analysis, authors proved, for example, that multiple-dose vitamin D administration was more effective than a single dose in the reduction in ICU admission (*p* = 0.044), the need for mechanical ventilation (*p* < 0.0001), and the length of hospitalization (*p* = 0.034) [186]. These results were further confirmed by Zhang X et al., who showed, in a meta-analysis of 21 studies, that vitamin D supplementation improved reduced mortality and ICU admission rates, particularly when administered continuously with a total dose of less than 100,000 IU over 14 days, among 25(OH)D-deficient (<30 ng/mL) COVID-19 patients but not in the non-restricted group [187].

Other meta-analyses have shown positive effects of vitamin D supplementation during COVID-19, although with partially conflicting results. For example, some have shown effects on COVID-19-associated mortality [188,189,190,191] and on the risk of admission to ICU [191,192], stressing the role of vitamin D deficiency in increasing the risk of infection [188], disease severity [188,190] and mortality [190]. Meanwhile, others proved no significance among the outcomes analyzed (e.g., mortality, length of hospital stay, ICU admission, need for mechanical ventilation) [192,193], although indicating an overall positive trend, especially in subjects receiving multiple vitamin D doses [193]. To date, no cases of vitamin D toxicity has been reported in its use to treat COVID-19.

## 4. Conclusions

In our narrative review, we aimed to analyze the non-pharmacological treatments most frequently used in patients who were not hospitalized because they had mild COVID-19. Among all the possible treatments, we focused only on the specific subset of common herbal products and dietary supplements that have been used in Italy, and, in particular, essential oils, ginseng, *Echinacea*, L-arginine, vitamin C, and, finally, vitamin D. However, it must be stressed that the quality and number of evidence supporting the use of the natural products are extremely different, as the use of vitamin supplements is much more consolidated than the use of herbal products (whether orally or by inhalation).

Although with contradictory results and based only on preliminary evidence, several in vitro and in vivo studies have shown that these molecules might shorten COVID-19 duration and might be effective in avoiding a severe clinical course, as well as the long COVID syndrome [194]. This appears to be related to the long persistence of immune system activation, as proven by the high levels of pro-inflammatory cytokines [195,196], which could be modulated by the activity of the natural products that we have described.

Due to the lack of strong evidence, herbal and dietary supplements should in no way be considered as a possible alternative to drugs approved for the treatment of SARS-CoV-2 infection, but only as a possible adjunctive therapy. In fact, they are not potent enough to replace an antiviral treatment, which, if administered within few days of infection, could be able to block SARS-CoV-2 replication, reducing the negative consequences of the cytokine storm, hence potentially decreasing COVID-19 clinical manifestations. Presently, early antiviral treatment represents the “standard of care” for patients, who do not need oxygen or mechanical ventilation. Herbal and dietary supplements might be useful as an adjunctive therapy in patients with mild disease and an indolent clinical course. In fact, even if confirmatory clinical trials are required, due to their anti-inflammatory, anti-thrombotic, and immune response modulation properties, they could potentially be a valid aid to prevent a severe clinical evolution, especially in co-morbid patients treated with other drugs. Moreover, among the potential benefits, which, however, require further evidence, vitamin D supplementation, or the administration of high-dose vitamin C plus L-arginine in hospitalized patients, could reduce the length of stay, the need for mechanical ventilation, and the death rate.

However, to date, very little is known about the concomitant use of multiple substances in SARS-CoV-2 infection, whether they are approved drugs or adjuvant therapies. This aspect would deserve to be explored with specific studies, first in vitro and then in vivo, specifically designed to understand the possible interactions between natural products and approved drugs.

Finally, no less important, more studies (e.g., large-scale randomized clinical trials) are required in the near future to standardize the potential non-pharmacological approach to SARS-CoV-2 infection.
